# The Genetic and Mechanistic Basis for Variation in Gene Regulation

**DOI:** 10.1371/journal.pgen.1004857

**Published:** 2015-01-08

**Authors:** Athma A. Pai, Jonathan K. Pritchard, Yoav Gilad

**Affiliations:** 1 Department of Biology, Massachusetts Institute of Technology, Cambridge, Massachusetts, United States of America; 2 Departments of Genetics and Biology, and Howard Hughes Medical Institute; Stanford University, Stanford, California, United States of America; 3 Department of Human Genetics, University of Chicago, Chicago, Illinois, United States of America; New York Genome Center, United States of America

## Abstract

It is now well established that noncoding regulatory variants play a central role in the genetics of common diseases and in evolution. However, until recently, we have known little about the mechanisms by which most regulatory variants act. For instance, what types of functional elements in DNA, RNA, or proteins are most often affected by regulatory variants? Which stages of gene regulation are typically altered? How can we predict which variants are most likely to impact regulation in a given cell type? Recent studies, in many cases using quantitative trait loci (QTL)-mapping approaches in cell lines or tissue samples, have provided us with considerable insight into the properties of genetic loci that have regulatory roles. Such studies have uncovered novel biochemical regulatory interactions and led to the identification of previously unrecognized regulatory mechanisms. We have learned that genetic variation is often directly associated with variation in regulatory activities (namely, we can map regulatory QTLs, not just expression QTLs [eQTLs]), and we have taken the first steps towards understanding the causal order of regulatory events (for example, the role of pioneer transcription factors). Yet, in most cases, we still do not know how to interpret overlapping combinations of regulatory interactions, and we are still far from being able to predict how variation in regulatory mechanisms is propagated through a chain of interactions to eventually result in changes in gene expression profiles.

## Introduction

Accumulating evidence indicates that gene regulatory changes often contribute to species-specific adaptations as well as to within-species variation in complex phenotypes [1], [2], such as interindividual variation in susceptibility to disease [3]–[5]. Indeed, motivated by theoretical arguments regarding the likely functional importance of variation in gene regulation and the emergence of genomic technologies that allow one to cheaply and rapidly characterize regulatory phenotypes, a large number of studies in the last decade have focused on uncovering the principles of gene regulation. These studies contributed to a rising recognition that natural variation in gene regulation may underlie most complex phenotypes within and between species. We have discovered a large number of regulatory mechanisms and described in detail many biochemical interactions that contribute to gene regulation. This has contributed to a better understanding of how regulatory information is encoded in the genome, and in a few cases, we have managed to manipulate gene regulatory programs and thereby affect complex phenotypes.

Yet, overall, we still have a limited ability to interpret how genetic variants alter gene regulation. We do not know how to “read the genome” and predict gene regulatory outputs. Our understanding of regulatory mechanisms and biochemical interactions has not yet matured into an ability to “read the code” and fully model transcriptional regulation.

Early studies of regulatory variation within and between species focused on characterizing steady-state mRNA levels, which represent the output of gene regulatory programs. For example, genome-wide comparative studies of steady-state mRNA levels were able to identify a large number of gene expression differences between species [6], [7]. However, while comparative studies facilitated the identification of interspecies regulatory differences that may be of functional importance, it was nearly impossible to pinpoint the genetic changes responsible for these differences. Thus, such studies had a limited ability to study the underlying molecular mechanisms of regulatory evolution.

In contrast to early comparative work, studies of mRNA levels within species were able to take the first steps towards the characterization of genetic variation in regulatory elements, even before the development of ultra-high-throughput sequencing technologies. This was done indirectly, using expression quantitative trait locus (eQTL) mapping to find associations between genotypes and variation in gene expression levels [8]–[10]. For most eQTLs the causal variant was unknown, and even when the likely causal variant could be inferred with relative confidence, the regulatory mechanism involved was generally difficult to identify [11]. Nevertheless, eQTL studies taught us about the spatial distribution of regulatory variants in the genome [11], the temporal specificity of the effect of regulatory sequences on expression patterns (namely, that some regulatory elements only affect gene expression under certain conditions), and the magnitude of steady-state expression changes associated with variation in *cis*- or *trans*-regulatory elements [12], [13].

With the rise of massively parallel sequencing technologies, both comparative studies of gene regulation and studies of regulatory variation within populations have been able to move beyond descriptions of steady-state mRNA expression levels [14], [15]. Recent studies have characterized interspecies and population-level variation in multiple aspects of gene regulation, including chromatin states [16], transcription factor (TF) binding footprints [17]–[20], profiles of different epigenetic markers [21]–[25], and posttranscriptional modifications [26]–[30]. These studies have been able to assess the correlation between variation in different regulatory mechanisms and variation in mRNA levels, as well as—using genotype data—infer the likely causal relationship between genetic variation, changes in regulatory interactions, and differences in gene expression levels. The combined analyses of data on multiple types of regulatory mechanisms often allow us to understand the basis for concerted changes in regulatory outputs, predict the consequences of a genetic change in regulatory sequences, and prioritize among statistically equivalent genetic associations of human diseases. Consequently, more complex models of regulatory interactions and their effects on gene expression have been developed.

Recent reviews have discussed the evolution of gene expression levels [14], the turnover in regulatory elements [31], and the insights from eQTL mapping studies [4], [32]. Here, we review recent insights into the genetic and mechanistic basis for variation in gene regulatory phenotypes, focusing especially on human studies. Our review concentrates on the efforts to perform combined analyses of multiple types of genomic data to obtain a more complete picture of the order of causal events that lead to precise gene regulatory programs. We focus particularly on mechanisms by which variants affect regulation of nearby genes (i.e., putatively in *cis*), as these are better understood and, moreover, likely represent the first step in most *trans*-acting QTLs as well. We examine the emerging models of causal relationships that explain concerted, or coordinated, changes in regulatory interactions and point to questions that are still unanswered regarding combinatorial relationships. Finally, we assess the proportion of variation in gene expression levels across individuals that could potentially be explained by variation in the regulatory mechanisms that have been studied thus far.

## Mapping Interindividual Variation in Gene Expression Levels

A surge of studies over the last few years have used eQTL mapping to identify substantial numbers of genetic variants affecting gene expression levels in humans across tissues, populations, and environmental or cellular conditions. One attractive property of eQTL mapping is the ability to infer a direct link between genotypic variation and phenotypic variation, such as differences in gene expression among individuals. Hence, eQTL mapping holds great promise as a method to annotate the function of regulatory loci throughout the genome and potentially identify causal genetic variants. Even using modest sample sizes (60–100 individuals), early studies found a large number of genetic associations with differences in gene regulation, identifying eQTLs for as many as 30% of genes in lymphoblastoid cell lines (LCLs) [12], [26], [33]. More recent studies, with larger sample sizes, have identified much larger numbers of eQTLs [34]–[36]. For instance, using RNA-sequencing-based expression data from whole-blood samples of 962 individuals, Battle et al. recently identified proximal (putatively *cis*-acting) eQTLs within 1 Mb of 78% of more than 10,000 tested protein-coding genes [36]. Consistent with earlier reports, the *cis* eQTLs found by Battle et al. were enriched near the 5′ ends of genes, suggesting that transcriptional regulation (rather than RNA decay) might be exerting the strongest amount of control on gene expression levels [11], [26], [33], [36].

The emerging pattern from recent eQTL studies, with sample sizes ranging from 1,000 to 5,000 individuals, is that virtually all expressed genes are likely to have at least one *cis*-acting eQTL (which can be detected if the sample size is large enough). Moreover, recent studies with large sample sizes have also started to achieve power to reliably identify *trans*-eQTLs, i.e., variants that affect the expression of both alleles of a gene; often the variants and the regulated genes are on different chromosomes [35], [36]. Heritability studies suggest that more than half of the genetically explained variance in gene expression is due to *trans*-acting variants [37], but reliable detection of *trans* eQTLs has been challenging in humans because the effect sizes of *trans*-acting variants tend to be smaller than for *cis* eQTLs [35], [36] and because there is a higher statistical penalty for multiple testing. One promising approach to overcome these issues might be to specifically focus on QTLs affecting the expression levels of putative *trans*-regulatory elements (thereby minimizing the number of tests performed). For instance, through possible *trans*-acting mechanisms that are still unclear, genetic variation affecting long intergenic noncoding RNAs (lincRNA) regulation could in turn be influencing the mRNA levels of subsets of protein-coding genes [38].

## Mapping Interindividual Variation in Gene Regulatory Mechanisms

**Figure 1:**
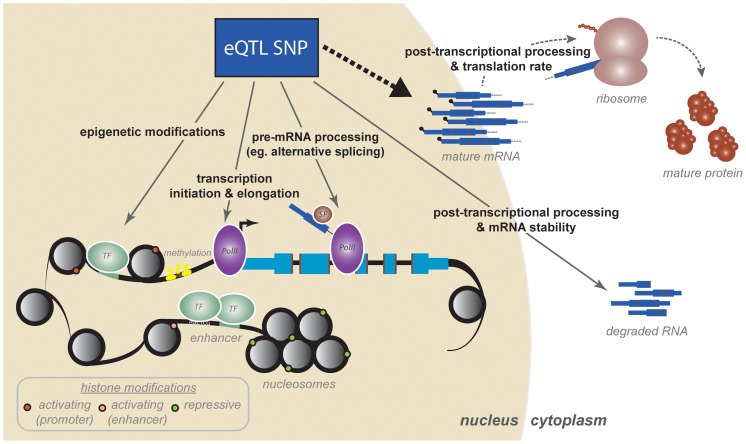
A cascade of regulatory mechanisms by which an eQTL SNP can affect gene expression. Studies mapping regulatory QTLs have identified a variety of mechanisms, many of which are coordinated, by which eQTLs might act to affect variation in mature mRNA levels. First, eQTL SNPs can impact epigenetic modifications and transcription initiation. These include regulatory processes such as transcription factor binding, histone modifications, enhancer activity (perhaps mediated by chromatin architecture and conformation), and DNA methylation. Transcriptional mechanisms, and specifically transcription factor binding, are likely the strongest contributors to variation in steady-state mRNA levels. Second, recent work has increased appreciation for transcriptional and cotranscriptional processes as major contributors to variation in gene expression levels and mRNA isoform diversity. These include mechanisms such as transcriptional elongation (by PolII traveling rates), cotranscriptional splicing, and mRNA processing and modification. Third, eQTL SNPs both within and outside the transcript have been shown to influence posttranscriptional mRNA processing, which includes mechanisms such as general mRNA degradation, defects in polyadenylation, and targeting by miRNAs. Finally, preliminary studies have shown that we do not yet fully appreciate the extent to which variation in mRNA expression might impact or even correlate to variation in downstream protein products, the synthesis of which are additionally regulated by a set of posttranscriptional and translational mechanisms.

Despite many attractive properties of the eQTL mapping approaches, merely mapping a locus associated with gene expression variation does not provide direct information about the mechanism perturbed by the associated genetic variant, even if one assumes that the causal variant has indeed been identified. To understand which regulatory mechanisms might be affected by eQTLs, the QTL mapping framework has been extended to consider a wide variety of genomic assays that relate to aspects of gene regulation. The rationale is that if an eQTL acts by perturbing a particular regulatory mechanism—for example, a histone modification—then the eQTL SNPs should also be associated with measures of the relevant regulatory mechanism(s). Such studies, which we will refer to as regulatory QTL (regQTL) studies, have yielded a number of intriguing insights into the mechanistic basis for eQTLs specifically and the complex and combinatorial nature of gene regulatory logic more generally (Fig. 1).

The paradigm of regQTL mapping has been applied within the context of numerous mechanisms spanning various stages of mRNA and protein regulation. The majority of regQTL studies have been conducted with sample sizes of fewer than 100 individuals, limiting their power to detect *trans* effects. We will thus focus on the insights gained from *cis*-acting regQTL maps. Moreover, with few notable exceptions [29], [30], [39], regQTL studies to date focus on mechanisms that regulate the rate of transcription and mostly neglect processes of posttranscriptional RNA processing and degradation. This is consistent with the prevalent notion that transcriptional mechanisms, as opposed to RNA decay, exert the largest control on gene expression phenotypes and might account for most of the observed variation in steady-state gene expression levels.

The analysis of eQTLs in the context of variation in regulatory mechanisms has generally involved correlating patterns across datasets collected from the same samples. This approach considers the genetic variation as a foundation for the purpose of inferring the causal order of events. It is indeed reasonable to assume that genetic diversity in a locus associated with regulatory variation is the initial cause for changes in regulatory mechanisms and, ultimately, in gene expression levels. However, inferring causality beyond the anchor of genetic diversity is more challenging and is generally done by identifying shared associations across multiple regulatory phenotypes. For example, genetic variants associated with dynamic epigenetic marks such as DNA methylation seem to contribute modestly to overall gene expression variation [21], [40]–[42]. In LCLs, an estimated 10%–20% of eQTLs are also classified as methylation QTLs (meQTLs) [21], and thus it could potentially be inferred that a small proportion of loci that are affecting gene expression do so by perturbing DNA methylation levels [21], [40], [41].

**Figure 2:**
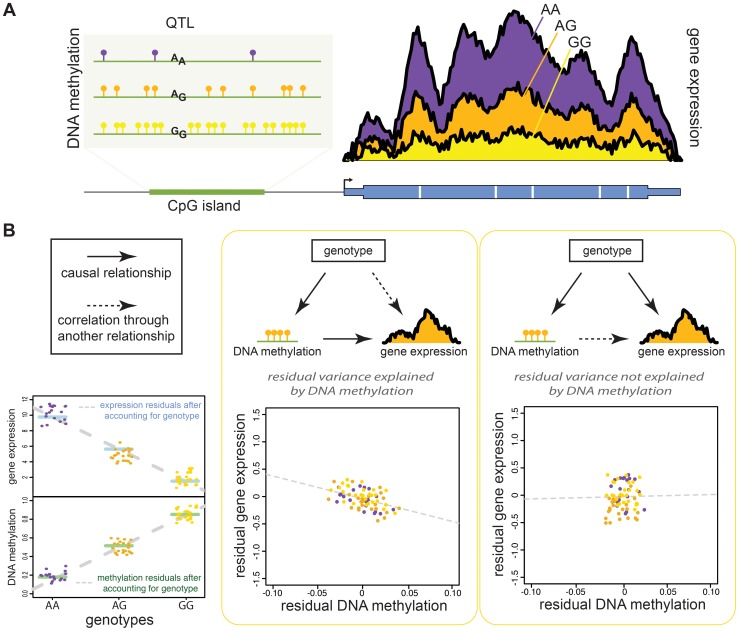
An approach for joint quantitative analysis of gene expression and regulatory QTLs. A goal of interindividual studies of regulatory mechanisms is to understand the extent to which variation at regulatory loci underlies gene expression levels across individuals. (A) This example, using hypothetical data, shows a QTL that is associated with levels of both DNA methylation in an upstream CpG island (left) and gene expression (right). Though the example QTL shown here indicates higher DNA methylation due to a G allele (potentially in a CpG pair), SNPs associated with methylation do not necessarily always fall in CpG dinucleotides. (B) The observed correlation between DNA methylation and gene expression levels could be due to a few different underlying relationships, two of which we have highlighted here. The extent to which gene expression and regulatory differences are correlated through an intermediate variable is often tested using an approach called partial correlation analysis. This involves regressing out the effects of an intermediate variable—genotype in this example—from both DNA methylation and gene expression levels and then evaluating the residual correlation between the two variables (left). One possibility is that the QTL directly affects differences in DNA methylation, which then determine (cause) the gene expression level. Thus, gene expression is regulated by the genotype through the DNA methylation effects (middle), and the residual variance in gene expression levels will still be correlated to residual DNA methylation levels. Alternatively, genotype is independently associated with both DNA methylation and gene expression levels—for instance, by directly influencing changes in an upstream mechanism (such as transcription factor binding) that affects DNA methylation and gene expression levels. This would make DNA methylation and gene expression appear to be correlated, but not causally related (right), and the residual values no longer show any significant correlation.

The inference of causality, however, is problematic. The intuitive interpretation of a SNP that is deemed to be both an eQTL and regQTL is that genetic variation at the QTL results in a change in a regulatory mechanism, which in turn results in a change of the expression of a nearby gene. Yet, the analysis of partial correlations across regulatory phenotypes does not generally indicate a straightforward sequence of molecular events (Fig. 2). In particular, it would seem that when the effects of genetic variation are accounted for, changes in gene expression levels and changes in epigenetic markers (methylation levels or histone modifications) are often not correlated with each other. This observation indicates that it is unlikely that there is a direct causal link between changes in the regulatory mechanism and differences in gene expression levels. Instead, an additional regulatory step may underlie the association of both gene expression and the epigenetic marker with genetic variation at the QTL.

One explanation for the observation of independent associations of genetic variation with regulatory marks may involve variation at TF binding sites. Indeed, variants influencing overall chromatin accessibility as measured by DNaseI hypersensitivity, which has long been used as a marker of regulatory activity in general and TF binding in particular, were found to overlap with as many as 55% of eQTLs in LCLs [19]. A joint analysis of gene expression, chromatin accessibility, and methylation reveals that DNaseI-sensitivity QTLs (dsQTLs) that also show significant association with methylation levels are more likely to be associated with gene expression changes. This observation may indicate that methylation, chromatin accessibility, and gene expression levels are either interacting or are all affected by changes in the same aspect of regulation—such as TF binding [19].

### Changes in transcription factor binding result in changes to the regulatory landscape

The suggestion that many of the genetic variants associated with variation in gene expression levels may do so by impacting TF binding affinity has recently gained some measure of support. A series of recent studies considered the regulatory impact of histone modifications, TF binding, and localization of RNA polymerase II (PolII) in small samples of individuals [16], [24], [25]. These complementary studies all found strong allele-specific signatures of PolII occupancy, histone modifications, and TF binding, consistent with earlier reports [17], [43]. Kasowski et al. used genome-segmentation methods (based on multiple histone modification profiles [44]) to understand the genetic basis of chromatin states. They found that enhancer states (defined primarily by H3K27ac and H3K4me1 histone modifications) exhibit the highest level of variability between individuals [16]. Yet, most QTLs associated with changes in enhancer-delineating histone modifications do not correspond to differences in gene expression levels. This may indicate that many apparent enhancers are nonfunctional; alternatively, there might be redundancy in enhancer function, absence of an intermediate component (such as a chromosome loop colocalizing the enhancer and promoter), compensatory effects, or buffering of transcript levels. Yet, it is also possible that interactions between histone modifications, which are implicitly assumed to be informative under the premise of annotating chromatin states [45], are less important as causal drivers of variation in gene expression levels than the marginal effects of either individual histone modifications or another underlying mechanism.

**Figure 3:**
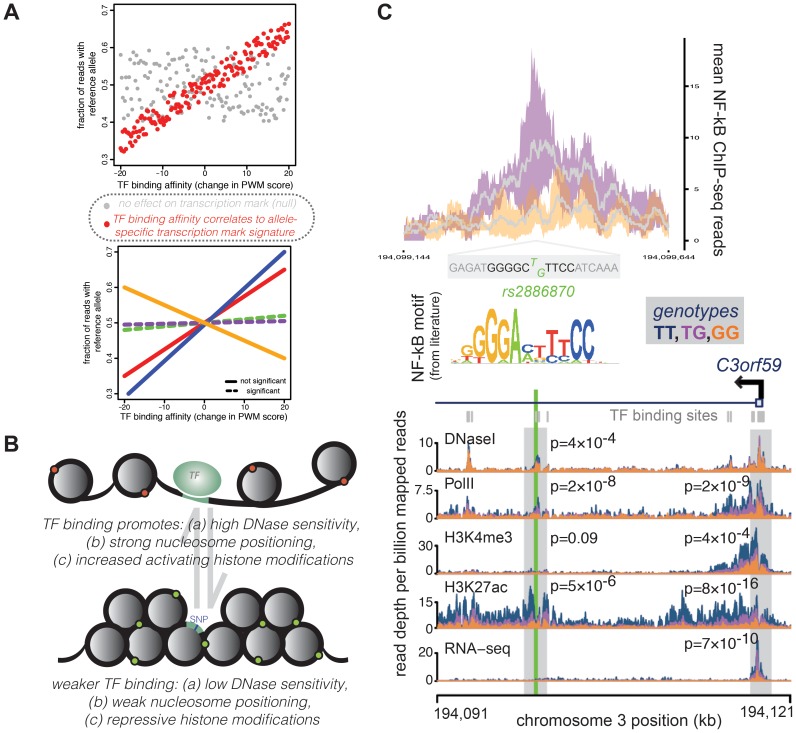
A representative example of a QTL in a TF binding site correlated with changes across multiple regulatory mechanisms. Many concerted changes in regulatory mechanisms across genotypes can be linked to a sequence change in transcription factor binding sites, which might causally influence downstream changes. (A) For TFs that regulate concerted changes in transcriptional marks, SNPs that cause a large change in binding affinity (as measured by a position weight matrix score, x-axes) might also show a large skew in the ratio of transcription mark reads from each allele (measured as fraction of reads from the reference allele, y-axes). Evidence for this correlation across all SNPs in binding sites (top panel, red points) implies a relationship between TF binding and the transcriptional mark. Significant correlations can then be assessed for a given TF across multiple transcriptional marks (bottom panel, where each line represents a correlation using allelic biases measured from different histone modifications, PolII localization, etc.) to understand which mechanisms might be influenced by changes in binding of the given TF. (B) Overall, looking at allelic biases in transcriptional marks at SNPs that can affect TF binding affinity show a pattern whereby increased TF binding is promoting open chromatin (measured by DNaseI sensitivity), nucleosome positioning, and enrichment of activating histone modifications relative to sites with weaker TF binding. Importantly, since SNPs in these binding sites usually only have moderate-to-weak effects on binding affinity, QTL SNPs most likely serve to shift the equilibrium frequencies between these two configurations within populations of cells. (C) This example of a TF binding QTL shows a SNP, rs2886870, that falls within a binding site for the NF-kB transcription factor. NF-kB ChIP-seq data [17] show that LCLs with at least one T allele (TG genotype; purple) matching the consensus motif sequence [63] have higher NF-kB binding than LCLs with no T alleles (GG genotype; orange). The top panel shows the distribution of ChIP-seq reads in a 500-bp window around rs2886870, with the grey line representing the mean across four individuals and the colored outlines representing the 95% confidence intervals. The rs2886870 SNP also acts as a QTL for several downstream regulatory patterns, with the T allele promoting significantly increased DNaseI hypersensitivity, PolII localization, and H3K7ac marks at the site of the transcription factor site and increased PolII localization, H3K4me3 marks, and H3K7ac marks at the promoter of the downstream C3orf59 gene, whose expression is also significantly associated with this QTL (panel reproduced from McVicker et al. 2013 [25]).

Kilpinen et al. and McVicker et al. focused more heavily on understanding the mechanistic basis of QTLs underlying individual histone modifications. By dissecting the strong links between histone modification QTLs (many of which regulated multiple histone modifications in addition to chromatin accessibility), both studies found that changes in sequence-based affinity for TF binding underlie a subset of observed changes in histone modifications and PolII occupancy across individuals (Fig. 3) [24], [25]. These studies propose that in some cases, TF binding is most likely the first step in a series of events that result in distinct histone modification profiles and gene expression output. Interestingly, by assaying nascent RNA expression levels in addition to processed mRNA expression levels, Kilpinen et al. were also able to observe greater evidence for allele-specific effects in nascent RNA than in mRNAs [24]. This observation points to additional and possibly complementary roles of posttranscriptional mechanisms that act in an allele-specific manner to influence steady-state gene expression levels.

An additional measure of support for the idea that TF binding may underlie general properties of chromatin states comes from a study that offered a different perspective on this challenging problem of inferring causality in the chain of regulatory processes. White and colleagues [46] used a massively parallel enhancer assay to test the activity of thousands of sequences. They tested 1,300 genomic sequences that were found, using high-throughput chromatin immunoprecipitation sequencing (ChIP-seq), to be bound in mouse retinas by the photoreceptor transcription factor Cone-rod homeobox (Crx). They also tested 3,000 control sequences, which were not bound by the TF but contained similar matches for the known binding motif of the TF. The enhancer assay was designed to address whether the bound sites are distinguishable from unbound sites by local primary sequence features or more influenced by the functional in vivo genomic context in which motifs reside, namely the chromatin and epigenetic context. The results were unambiguous: in the vast majority of cases, the sequence information in segments of just 84 bp centered in individual ChIP-seq peaks was sufficient to distinguish between bound and unbound sites [46]. These observations further support the notion that TF binding is primarily determined by the sequence context and is less driven by chromatin state. That said, it will be important to repeat these experiments in more cell types, with more factors, in order to understand the generality of these results.

### The genetic basis of variation in posttranscriptional regulatory mechanism

There have been far fewer studies that focused on the extent to which eQTLs are driven by posttranscriptional regulatory mechanisms, perhaps due to the more complex technical challenges of assaying posttranscriptional mechanisms on a large scale. Several studies have mapped the genetic basis of mRNA splicing variation (splicing QTLs [sQTLs]) and found that much of the variation in splicing is located within or proximal to the targeted spliced exon [26], [36], [47]. As expected, many sQTLs fall directly within primary splice sites. However, sQTLs also unexpectedly show a prominent enrichment near transcription start sites and 5′ untranslated regions (UTRs) and within TF binding sites [36]. This might suggest that splicing mechanisms could be either independently or concurrently regulating gene expression through interactions with components of the transcriptional regulatory machinery.

A small number of studies have also investigated the role of other posttranscriptional mechanisms, such as general RNA decay (RNA decay [rdQTLs]) [30], alternative polyadenylation [29], and miRNA binding (miRNA binding QTLs [mirQTLs]) [34], [39], [48]. Each of these QTL types shows enrichment in 3′ UTR motifs or regulatory elements that have previously been implicated in transcript stability, such as adenylate-uridylate (AU)-rich elements and miRNA binding sites [29], [30], [39]. Interestingly, rdQTLs and mirQTLs are also often associated with variation in mRNA expression levels [30], [39], [48]. SNPs associated with miRNAs tend to affect miRNA biogenesis rather than target recognition within the transcript, and stronger regulation by miRNAs is associated with greater variation in gene expression levels [39]. However, the picture presented by rdQTLs is more complex, with almost half of the nearly 200 rdQTLs identified showing counterintuitive associations with mRNA expression levels (namely, the allele associated with more rapid RNA decay is also associated with higher levels of steady-state expression). The mechanisms underlying these opposite-direction effects are unclear, but they might reflect a buffering mechanism or a coupling between transcription and decay [49]. Overall, it was estimated that as many as 19% of eQTLs might be driven by differences in mRNA decay [30].

There is still much to be learned about transcriptional and posttranscriptional mechanisms. Yet, it is also becoming clear that in order to fully appreciate gene regulatory effects on human phenotypes, one would have to incorporate studies of protein expression levels [50]–[52]. In the first study of its kind in humans, Wu et al. studied the genetic basis of protein expression levels in LCLs and the effects of mRNA variation on downstream protein level phenotypes [50]. They found considerable interindividual variation in protein levels across the 95 LCLs they investigated and were able to identify 77 protein QTLs (pQTLs). Only about half of these pQTLs were also found to be affecting transcript levels, which may indicate that many pQTLs specifically affect the regulation of translation or protein stability. Perhaps more interesting is the observation that most (more than 80%) of the eQTL SNPs found in these LCLs were not also associated with variation in protein levels [50]. This discrepancy might be partly explained by incomplete power to detect pQTLs, perhaps due in part to limitations of the mass spectrometry techniques for protein measurements. Yet, these observations are also consistent with the results of a recent comparative proteomics study [53], which found evidence consistent with buffering or compensation of interspecies divergence in protein expression levels.

### Can we explain variation in gene expression?

Posttranscriptional mechanisms notwithstanding, the available body of work suggests that variation in steady-state gene expression levels can disproportionately be explained by variation in TF binding. In turn, variation in TF binding might underlie concerted changes in a large number of supporting regulatory mechanisms that determine chromatin state and accessibility [19], [24], [25]. These inferences, based on functional genomic variation data, are consistent with independently observed evidence of strong selective pressures on TF binding motifs, second only to the conservation of protein-coding genes [54]. It would thus seem that we have made considerable steps towards an understanding of important properties of gene regulatory logic and the ability to explain variation in gene expression.

Yet, one ultimate goal of genomics is to read the code—to be able to predict variation in gene expression levels based on the nucleotide sequence—and this goal remains challenging. The main difficulty is that many changes in TF binding do not seem to result in measurable changes in gene expression levels, and we do not yet know how to distinguish between binding events that affect gene expression and those that do not [55]. Perhaps much of TF binding is not directly functional, or maybe the marginal effects of a change in binding of one TF are too small to detect given the complexity of interactions between different regulatory mechanisms.

For example, Cusanovich and colleagues [55] attempted to characterize functional TF binding by characterizing genome-wide gene expression profiles following the independent knockdowns of 59 TFs in the same lymphoblastoid cell line. Depending on which TF was knocked down, the expression levels of a few dozen to several thousands of genes were significantly perturbed. However, in all cases, only a small subset of genes inferred to be bound by any individual TF were differentially expressed following the knockdown of that factor [55]. This observation suggests that many instances of TF binding in the genome do not result in measurable changes in gene expression levels of the putative target genes.

Regardless of the logic of functional TF binding, while transcriptional regulation at promoters and enhancers outweigh known posttranscriptional effects, it is clear that not all regulation is happening within promoter regions. Along these lines, many interesting nonintuitive interactions between regulatory elements have emerged from regQTL studies. For instance, both sQTLs and rdQTLs, which are primarily enriched within canonical splice site motifs and 3′ UTR stability motifs, respectively, also have strong signals at the transcription start site and seem to be cooperatively regulating gene expression variation with transcriptional mechanisms [30], [36]. These observations highlight the fact that none of these mechanisms work in isolation and complex coregulatory phenomena are quite common, if likely situation dependent. Similarly, though regQTL studies have advanced our understanding of the mechanistic basis for many eQTLs, there remain many regQTLs that seem to have no discernable effect on gene expression phenotypes [19], [21], [30]—for instance, while 55% of eQTL SNPs are identified as dsQTLs, only 39% of all dsQTLs are also associated with changes in gene expression levels [19]. Combined studies of quantitative posttranscriptional and protein measurements might provide insight into many of these non-eQTL regQTLs, but overall, the functional consequences of these unexplained regQTLs remains an unanswered question in the field.

## What Have We Learned and What's Next?

While recent work analyzing combinations of functional genomic data types has taught us much about the principles of gene regulatory logic, the results have more importantly opened the door for new sets of questions to be addressed. Theoretical models of gene regulatory networks and logic can finally be tested and refined based on directly relevant genomic data of high resolution and incredible breadth and depth. Outstanding questions about causality, the order of regulatory events, and the direction of effects can finally be addressed. For instance, the initial intuition that histone modifications regulate chromatin state, which in turn determine whether factors can bind to different sites, might generally be inaccurate [24], [25], [56]. Instead, TF binding seems to be the central event that mediates concerted changes in other regulatory mechanisms determining chromatin states, accessibility, and conformations. This, however, cannot be an exclusive statement because it would require that the entire system rely entirely on the variation in TF expression. An intermediate model has been suggested, by which a small number of particular TF, at a subset of their designated binding sites, can act as pioneer factors. Pioneer activity generates concerted changes in chromatin state, which are maintained by histone marks, DNA methylation, and nucleosome positioning [57]–[59]. Chromatin areas that are accessible because of pioneer activity are available for binding by secondary factors [57], [58]. Though direct evidence for the model of pioneer transcription factors is still weak, the model is consistent with all of the genomic variation data collected to date.

One promising way to move forward is by considering a combination of data across individuals and across tissue types (for example, the Genotype-Tissue Expression [GTEx] study). While the genetic and mechanistic basis for regulatory variation across individuals is of great interest, one of the factors limiting the utility of population data is that relatively modest regulatory difference are observed between individuals. Regulatory differences between tissues or different cell types are of a much larger magnitude. Moreover, while the regulatory landscape in the same cell type or tissue across individuals is highly similar (with the occasional difference due to genetic or epigenetic variation), the regulatory programs in different cell types can be vastly different. It is thus expected that a combined analysis of regulatory variation between individuals and across tissues may have more power to detect partial correlations and thus infer causality.

With a better understanding of the cascade of regulatory events that leads to variation in gene expression outputs, we can turn our attention back to the persistent questions that motivate much of the research in the fields of regulatory evolution and disease susceptibility. What are the modes of evolution within species? How many regulatory changes underlie a phenotypic adaptation and what mechanisms are affected? What are the most important genetic changes in the evolution of particular lineages?

The genomic tools that allow us to collect appropriate data with which to address these questions are already largely available. For example, it is now possible to perform genome editing of specific loci in order to specifically test the causal role of individual nucleotide changes. Yet, in humans—where direct manipulation of the entire individual is not possible—a suitable and faithful cellular system is needed in order to carry out such experiments. Studies using the LCL system in humans have yielded a wealth of information and insight as we considered steady-state regulatory phenotypes, but it seems that we have nearly exhausted the usefulness of this artificial system, which does not lend itself well to temporal, developmental, or spatial variation in gene regulation. New systems, such as induced pluripotent cells (iPSC) and their derived differentiated cell types, are perhaps a more appropriate and fertile resource for such studies [60]–[62].
